# Identification of *N*^*α*^-acetyl-*α*-lysine as a probable thermolyte and its accumulation mechanism in *Salinicoccus halodurans* H3B36

**DOI:** 10.1038/srep18518

**Published:** 2015-12-21

**Authors:** Kai Jiang, Yanfen Xue, Yanhe Ma

**Affiliations:** 1 State Key Laboratory of Microbial Resources and National Engineering Laboratory for Industrial Enzymes, Institute of Microbiology, Chinese Academy of Sciences, Beijing, China; 2 University of Chinese Academy of Sciences, Beijing, China

## Abstract

*Salinicoccus halodurans* H3B36 is a moderate halophile that was isolated from a 3.2-m-deep sediment sample in Qaidam Basin, China. Our results suggest that *N*^*α*^-acetyl-*α*-lysine can accumulate and act as a probable thermolyte in this strain. The accumulation mechanism and biosynthetic pathway for this rare compatible solute were also elucidated. We confirmed that the *de novo* synthesis pathway of *N*^*α*^-acetyl-*α*-lysine in this strain starts from aspartate and passes through lysine. Through RNA sequencing, we also found an 8-gene cluster (orf_1582–1589) and another gene (orf_2472) that might encode the biosynthesis of *N*^*α*^-acetyl-*α*-lysine in *S. halodurans* H3B36. Orf_192, orf_193, and orf_1259 might participate in the transportation of precursors for generating *N*^*α*^-acetyl-*α*-lysine under the heat stress. The transcriptome reported here also generated a global view of heat-induced changes and yielded clues for studying the regulation of *N*^*α*^-acetyl-*α*-lysine accumulation. Heat stress triggered a global transcriptional disturbance and generated a series of actions to adapt the strain to heat stress. Furthermore, the transcriptomic results showed that the regulon of RpoN (orf_2534) may be critical to conferring heat stress tolerance and survival to *S. halodurans*.

Organisms have evolved a series of protective mechanisms to cope with the challenges of a changing environment. The most widely used strategy is the accumulation of large quantities of organic protectants, known as compatible solutes, and the switching of their type or concentration according to differing environmental conditions, such as temperature or salt^1^,^2^,^3^,^4^. The term “compatible solute” refers to low-molecular-weight, water-soluble, organic compounds that the cell can accumulate through *de novo* synthesis or external uptake in exceedingly high concentrations under extreme conditions, such as osmotic, heat or cold stress, without affecting enzyme activity or cell metabolism^5^,^6^. Previous researches have shown that different classes of chemical compounds, such as sugars, sugar derivatives, polyols, phosphodiesters, and amino acids, and their derivatives can act as compatible solutes^7^,^8^,^9^,^10^.

Among these known osmolytes and thermolytes, the lysine derivatives are a compatible solute type of particular interest. Recently, clues have arisen to suggest a connection between lysine catabolism and stress protection^11^. Lysine is converted into α-aminoadipic-δ-semialdehyde (AASA), a physiological intermediate that participates in stress response^12^,^13^,^14^,^15^,^16^,^17^. The compatible solute pipecolate or its derivatives can be converted from AASA and accumulate to high levels to play a role in salt resistance^11^,^17^,^18^,^19^,^20^,^21^. Some *N*-acetylated lysine, such as *N*^*ε*^-acetyl-*β*-lysine, *N*^*ε*^-acetyl-*α*-lysine and *N*^*α*^-acetyl-*α*-lysine, can also serve as cytoprotectants in a number of microorganisms. *N*-acetylation can transform positively charged lysine into neutral zwitterionic molecules and render it more water-soluble. The synthesis of *N*^*ε*^-acetyl-*β*-lysine is widely distributed and has been found in methanogenic Archaea, *Bacillus cereus* CECT 148 and several strains of green sulphur bacteria^7^,^8^,^22^,^23^. The synthesis pathway of this metabolite has already been elucidated and involves the conversion of lysine through the action of lysine-2,3-aminomutase and β-lysine acetyltransferase^24^,^25^. *N*^*ε*^-acetyl-*α*-lysine, which was produced via an acetyltransferase, can accumulate in *Planococcus* sp. VITP21 and a strain of *Salinicoccus hispanicus*^26^,^27^,^28^. Until recently, *N*^*α*^-acetyl-*α*-lysine has only been found in *Salinibacter ruber* in low concentrations, where it contributed to the osmotic balance^29^. *N*^*α*^-acetyl-*α*-lysine is a less-characterised compound and the synthesis pathway and the mechanism of *N*^*α*^-acetyl-*α*-lysine accumulation are unknown.

In the current study, we found that *Salinicoccus halodurans* H3B36, which was isolated from a sediment sample (3.2 m deep) in the Qaidam Basin, can accumulate *N*^*α*^-acetyl-*α*-lysine acting as an organic cytoprotectant. This substance exhibited heat stress-relieving properties, and its concentration increased by several fold when the cultivation temperature was raised. C^13^-labelling experiments and transcriptomic analysis were employed to explore the *N*^*α*^-acetyl-*α*-lysine accumulative mechanism and gain a broad understanding of the molecular basis of heat acclimation in *S. halodurans* H3B36.

## Results and Discussion

### Detection of organic osmolytes and identification of the unknown compatible solute in *S. halodurans* H3B36

**Figure 1:**
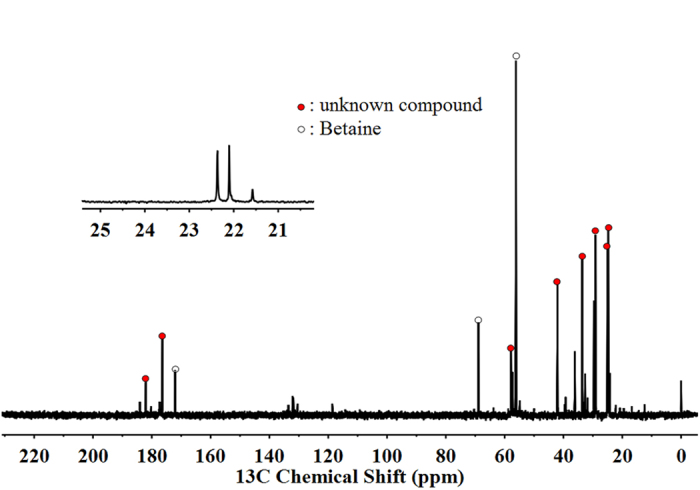
Natural abundance ^13^C spectrum (recorded in D_2_O as the solvent) of the cell extract of *S. halodurans* H3B36 grown at 6% NaCl (w/v). The chemical shifts of betaine were δ172.08, 68.92, 56.20, 56.17, and 56.14, and the chemical shifts of the unknown compound were δ182.06, 176.40, 57.84, 42.05, 33.64, 29.10, 25.02, and 24.75. The paramagnetic ions present in the sample caused a small shift in the signals compared with the values in the literature. TMSP was used as the internal reference.

We used high-performance liquid chromatography (HPLC) to analyse the organic osmolytes of *S. halodurans* H3B36 under growth in medium GMH with 6.0% NaCl (w/v). Commercially available trehalose, sucrose, glycine betaine, ectoine, hydroxyectoine, proline, alanine and glucose were chosen as standards to represent a spectrum of osmolytes. ^13^C- nuclear magnetic resonance spectroscopy (NMR) was also used to overcome the limitations of HPLC and ensure the complete capture of the organic cellular solute pool. According to the retention times of standard substances monitored by photodiode array detector (DAD) and refractive index detector (RID) and the related chemical shifts in the natural abundance ^13^C-NMR spectrum, glycine betaine and an unknown osmoprotectant were found to be accumulated in strain H3B36. The natural abundance ^13^C-NMR spectrum of *S. halodurans* H3B36 cell extract is shown in Fig. 1.

To identify the unknown compatible solute, we purified this compound and examined it using LC-MS and NMR to determine its molecular formula and structure. A molecular peak (M + H) + at m/z 189.1238 was discovered by applying high-resolution ESI-MS to the purified compound. Therefore, its molecular formula was proposed to be C_8_H_16_N_2_O_3_. This presumption was confirmed by elemental analysis, which suggested a C:H:N ratio of 4:8:1. The experimental ^13^C-NMR, ^1^H-NMR and ^1^H-^13^C-HMBC spectra were shown in supplementary Fig. S1- Fig.S3 online, respectively. The one-dimensional NMR spectrum of the sample revealed the presence of at least one CH_3_, three CH_2_, one CH and two carboxyl groups. Furthermore, the protons in the CH_3_ group did not couple with other protons. Therefore, the compound was either *N*^*α*^-acetyl-*α*-lysine or *N*^*ε*^*-*acetyl*-α-*lysine. The HMBC experiment, which can provide the correlation between ^1^H and ^13^C shifts (within 2 to 3 bonds), showed that the protons in the α-position (CH shift at 4.15 ppm) have connections with both the carbonyl of the acetyl group (^13^C shift at 176.43 ppm) and the carboxyl group (^13^C shift at 181.98 ppm). This finding suggested that the two carboxyl groups are closed. Based on these results, the compound was identified as *N*^*α*^-acetyl-*α*-lysine. This conclusion was confirmed by experiments using *N*^*α*^-acetyl-*α*-lysine purchased from Sigma (USA).

### Effect of salinity and heat stress on the accumulation of *N* ^*α*^-acetyl-*α*-lysine in *S. halodurans* H3B36

**Figure 2:**
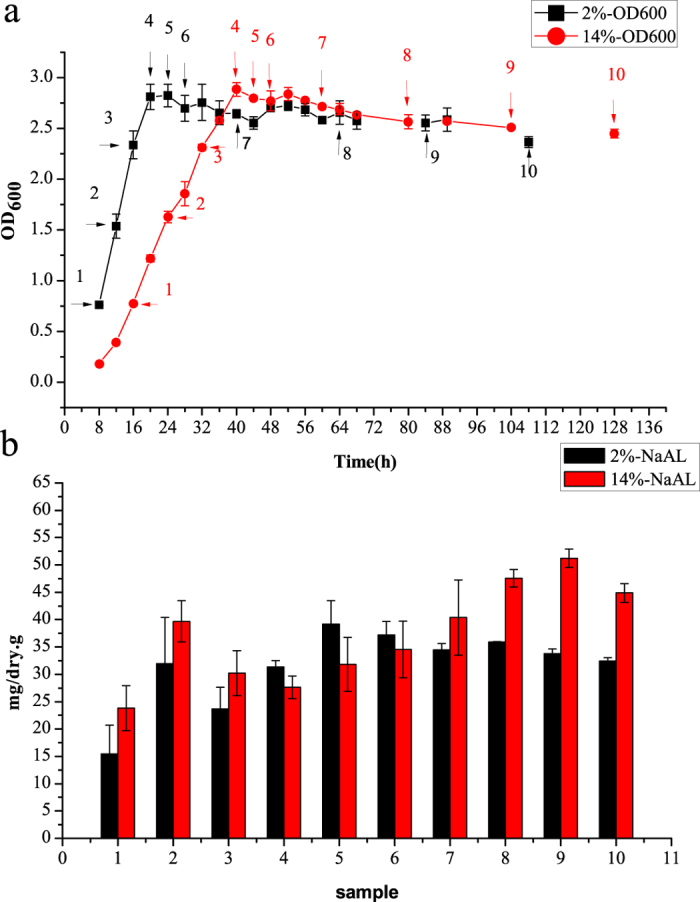
Effect of NaCl concentration on the accumulation of *N*^*α*^-acetyl-*α*-lysine in *S. halodurans* H3B36. (**a**) Growth curves and sampling points for *S. halodurans* H3B36 cells cultured in GMH medium containing 2% or 14% NaCl at 30 °C and pH 7.0; 2%-OD600 and 14%-OD600 represent the OD_600_ of the cells cultured at 2% and 14% NaCl, respectively. (**b**) The concentration of *N*^*α*^-acetyl-*α*-lysine accumulated in *S. halodurans* H3B36 at every corresponding sampling point. Intracellular amounts of *N*^*α*^-acetyl-*α*-lysine were calculated based on cellular dry weight; 2%-NaAL and 14%-NaAL represent the intracellular amounts of *N*^*α*^-acetyl-*α*-lysine at 2% and 14% NaCl, respectively.

**Figure 3:**
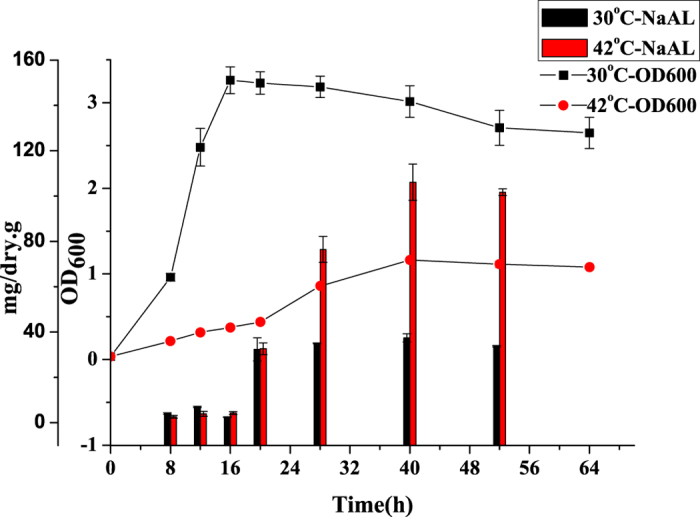
Growth curves and intracellular amounts of *N*^*α*^-acetyl-*α*-lysine of *S. halodurans* H3B36 grew in liquid GMH medium with 6% NaCl at different growth temperatures. Intracellular amounts of *N*^*α*^-acetyl-*α*-lysine were calculated based on cellular dry weight. 30 °C-OD600 and 42 °C-OD600 represent the OD_600_ of the cells cultured at 30 °C and 42 °C, respectively. 30 °C- NaAL and 42 °C- NaAL represent the amount of *N*^*α*^-acetyl-*α*-lysine of cells cultured at 30 °C and 42 °C, respectively.

Strain H3B36 was able to grow at salinities of 2–18% (w/v) NaCl (optimally at 4–6%, w/v) and at 4–42 °C (optimally at 30 °C) in GMH medium. This strain did not grow in medium without salt, nor did it grow well with high levels of salt, in non-enriched medium, or below 16 °C. Taking extraction efficiency and repeatability into consideration, the amount of *N*^*α*^-acetyl-*α*-lysine was calculated based on cellular dry weight. We chose 2% and 14% NaCl (w/v) to test the effect of salinity on *N*^*α*^-acetyl-*α*-lysine accumulation in *S. halodurans* H3B36. As shown in Fig. 2, there was no significant difference in the level of *N*^*α*^-acetyl-*α*-lysine accumulation when strain H3B36 was cultured in GMH with 2% NaCl (w/v) and 14% NaCl (w/v). The effect of heat on the accumulation of *N*^*α*^-acetyl-*α*-lysine was examined at 30 °C and 42 °C (Fig. 3). HPLC quantification results showed that the accumulation of *N*^*α*^-acetyl-*α*-lysine increased over time and reached its highest level in the stationary phase. The amount of *N*^*α*^-acetyl-*α*-lysine accumulated in the cytoplasm at 42 °C was two-fold more than at 30 °C. Higher temperatures were a challenge for the growth of this strain. An increase of 12 °C above the optimal temperature clearly impaired the final cell density (the OD_600_ decreased from 3.19 to 1.16). *N*^*α*^-acetyl-*α*-lysine may be accumulated to cope with this threat. Glycine betaine also existed in the solute pool of *S. halodurans* H3B36. The amount of Glycine betaine accumulated rapidly and reached at a high level in the early exponential phase, and then decreased along with the growth. The trend of decline of glycine betaine accumulated in cell had not changed when cultured at 42 °C. However, the accumulation of glycine betaine at 42 °C was slightly higher than at 30 °C and the rate of decline of glycine betaine at 42 °C was slower than at 30 °C (see Supplementary Fig. S4 online).

Some compatible solutes possess excellent thermal stress-relieving properties in the intracellular environment. For example, di-*myo*-inositol phosphate and mannosylglycerate are two thermolytes that are abundant in many thermophiles and hyperthermophiles^4^,^30^. Trehalose is considered a universal stress molecule and plays a key role in thermotolerance in bacteria, yeasts, fungi, archaea and plants^31^,^32^, and 5-hyroxyectione is produced in response to high temperatures in some halophilic or halotolerant *Actinobacteridae*, *Firmicutes* and *Proteobacteria*^33^,^34^. Glycine betaine acts as a thermoprotectant in various microorganisms, such as *Escherichia coli* and *Bacillus subtilis*^35^,^36^. Our experiments indicated that *N*^*α*^-acetyl-*α*-lysine, which is a rare and less-characterised compatible solute, acts as a probable thermolyte in *S. halodurans* H3B36, and glycine betaine has slight thermo-protection in *S. halodurans* H3B36 too. Our result was the first evidence of *N*^*α*^-acetyl-*α*-lysine accumulation under heat stress.

### Accumulative mechanism and metabolic pathway of *N* ^*α*^-acetyl-*α*-lysine in *S. halodurans* H3B36

Microorganisms can accumulate compatible solutes through the uptake from the environment or *de novo* through the synthesis^6^,^7^,^8^,^9^,^10^. Where did *N*^*α*^-acetyl-*α*-lysine come from? For solving this question, we used synthetic medium MM to replace semi-defined medium GMH that contains Yeast extract. Although *S. halodurans* H3B36 did not grow well in medium MM, it still reached a final OD_600_ of approximately 0.3. We collected the cells cultivated in medium MM, examined their compatible solute pool and found that *N*^*α*^-acetyl-*α*-lysine was present. These clues suggest the *de novo* synthesis of *N*^*α*^-acetyl-*α*-lysine exists in *S. halodurans* H3B36.

**Figure 4:**
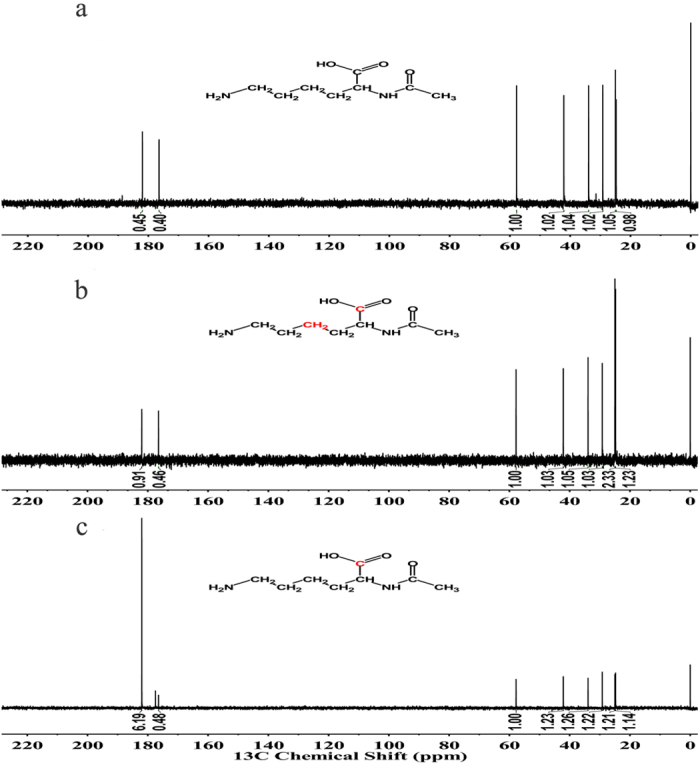
^13^C-NMR spectra of purified *N*^*α*^-acetyl-*α*-lysine from *S. halodurans* H3B36. (**a**) Natural abundance; (**b**) growth with 5 mmol/L ^13^C-labelled aspartic acid; (**c**) growth with 5 mmol/L ^13^C-labelled lysine. Red represents the ^13^C-labelled carbon in the molecular formula. TMSP was used as the internal reference.

That raises a question about what the *de novo* pathway and the precursor of *N*^*α*^-acetyl*-α-*lysine are. Some clues have been given from the pathway of other lysine derivatives and the structural analogues of *N*^*α*^-acetyl*-α-*lysine (see Supplementary Fig. S5 online). *N*^*ε*^-acetyl-*α*-lysine can generate from lysine directly and *N*^*ε*^-acetyl-*β*-lysine can also use lysine as precursor. Acetyltransferases are the key enzymes in these processes^24^,^25^,^26^,^27^,^28^. LysW-γ-lysine and *N*^*α*^-acetyl-ornithine are the structural analogues of *N*^*α*^-acetyl*-α-*lysine. The pathways of the two compounds have something in common : (1) direct modification of the –NH_2_ group of the corresponding substrates at the α-position at the beginning of the reaction; (2) using kinase and reductase to generate intermediates of semialdehyde-type; (3) production of the final compounds through a amino transfer reaction^37^,^38^,^39^,^40^,^41^,^42^,^43^. To investigate the accumulative mechanism and biosynthetic pathway in *S. halodurans* H3B36, we introduced two specific ^13^C-labelled substrates into the medium. As showed in Fig. 4, when cells were grown in the presence of [1-^13^C] lysine, the ^13^C-NMR spectrum of *N*^*α*^-acetyl-*α*-lysine resulted in one labelled carbon. The result suggests *S. halodurans* H3B36 can synthesise *N*^*α*^-acetyl-*α*-lysine from lysine directly. Therefore, the biosynthetic pathway of *N*^*α*^-acetyl-*α*-lysine in *S. halodurans* is similar to that of *N*^*ε*^-acetyl-*α*-lysine and *N*^*ε*^-acetyl-*β*-lysine, and an acetyltransferase might play a key role in this process. Such short biosynthetic pathway from lysine is helpful for the application of *N*^*α*^-acetyl-*α*-lysine. Additionally, when cells were grown in the presence of [1,4-^13^C] aspartate, the ^13^C-NMR spectrum clearly showed that *N*^*α*^-acetyl-*α*-lysine is also labelled. This result suggests that *N*^*α*^-acetyl-*α*-lysine can be biosynthesised from aspartate, too.

**Figure 5:**
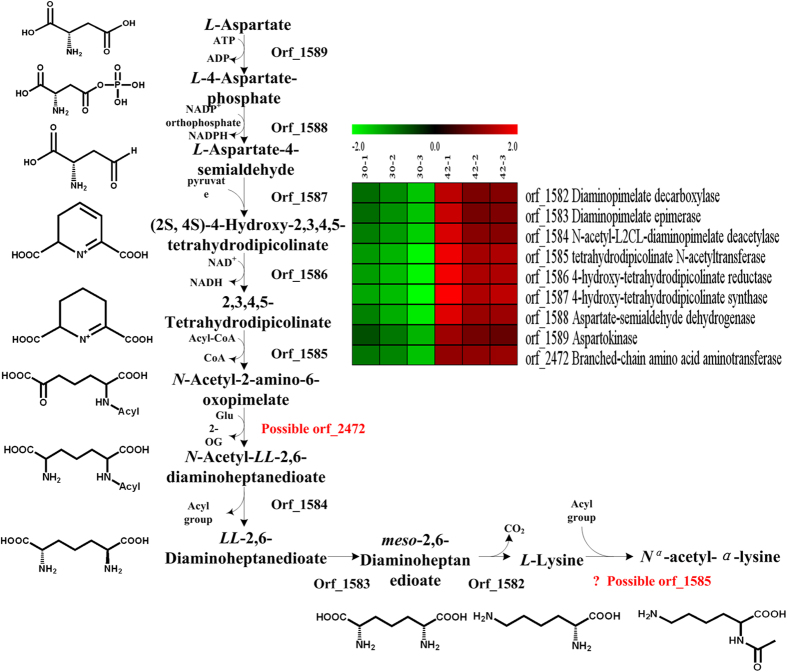
The decuced pathway of *N*^*α*^-acetyl-*α*-lysine and the differential transcription of putative genes involved in *N*^*α*^-acetyl-*α*-lysine biosynthesis in *S. halodurans* H3B36. The chemical structures of intermediates are shown beside. The expression profiles are presented based on log_2_FPKM. Red, upregulation. Green, downregulation. 30-1, 30-2 and 30-3 represent three duplicates originating from the 30 °C cultures; 42-1, 42-2 and 42-3 represent three duplicates originating from the 42 °C cultures.

The complete genome sequence of *S. halodurans* H3B36 had been deposited in GenBank under accession number CP011366. KEGG pathway analysis of the genome showed that *N*^*α*^-acetyl-*α*-lysine synthesized from aspartate may be generated through the acetyl-dependent diaminopimelic acid pathway (DAP) in *S. halodurans* H3B36 (Fig. 5). Based on sequence homology, an 8-kb cluster containing 8 genes (orf_1582-orf_1589) was predicted to be involved in *N*^*α*^-acetyl-*α*-lysine biosynthesis. Orf_1589 likely encodes an aspartokinase that shares 42.32% sequence identity with the homologous gene in *Bacillus subtilis* (Swiss-Prot geneID: AK2_BACSU); orf_1588 may encode an aspartate-semialdehyde dehydrogenase that shares 49.37% sequence identity with the homologous gene in *Aquifex aeolicus* (Swiss-Prot geneID: DHAS_AQUAE); orf_1587 may encode a dihydrodipicolinate synthase that shares 54.79% sequence identity with the homologous gene in *Staphylococcus aureus* (Swiss-Prot geneID: DAPA_STAAT); orf_1586 may encode a dihydrodipicolinate reductase that shares 52.92% sequence identity with the homologous gene in *Staphylococcus carnosus* (Swiss-Prot geneID: DAPB_STACT); orf_1585 may encode a 2,3,4,5-tetrahydropyridine-2,6-dicarboxylate N-acetyltransferase that shares 70.17% sequence identity with the homologous gene in *Staphylococcus haemolyticus* (Swiss-Prot geneID: DAPH_STAHJ); orf_1582 may encode a diaminopimelate decarboxylase that shares 55.61% sequence identity with the homologous gene in *Staphylococcus aureus* (Swiss-Prot geneID: DCDA_STAAC). The products of these six genes are all related to lysine biosynthesis enzymes. Orf_1584 and orf_1583, the remaining two genes of the gene cluster, share 60.32% sequence identity with the uncharacterised hydrolase SSP1352 from *Staphylococcus saprophyticus* (Swiss-Prot geneID:Y1352_STAS1) and 53.65% sequence identity with the alanine racemase from *Staphylococcus aureus* (Swiss-Prot geneID: ALR2_STAAC), respectively, and partially match the N-acetyldiaminopimelate deacetylase and diaminopimelate epimerase, respectively, in the DAP pathway.

### Transcriptional changes in *S. halodurans* H3B36 in response to heat shock

**Figure 6:**
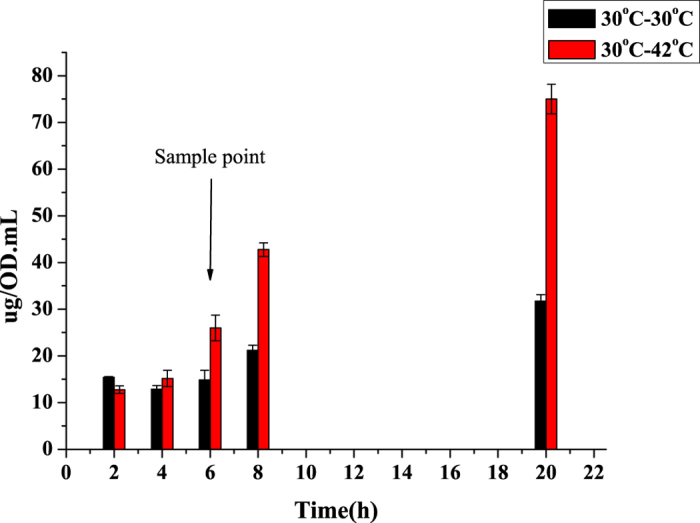
Heat shock experiment (measure intracellular amounts of *N*^*α*^-acetyl-*α*-lysine at different times after subculturing mid-exponential phase cells to 42 °C) and sample point for RNA sequencing. The culture of *S. halodurans* H3B36, which cultured in GMH medium containing 6% (w/v) NaCl at 30 °C, was split and shaken at 30 °C and 42 °C separately after reached to mid-exponential phase (an OD_600_ range of 0.5 to 0.6). The intracellular *N*^*α*^-acetyl-*α*-lysine concentrations of cultures were begun to measure at different time (2, 4, 6, 8, or 20 h) after heat shock. Intracellular amounts of *N*^*α*^-acetyl-*α*-lysine were calculated based on OD_600_ (i.e., μg per 1 mL per 1 OD_600_); (30 °C−30 °C)-NaAL represents subculturing to 30 °C (the control), and (30 °C−42 °C)-NaAL represents subculturing to 42 °C. The sample point marked in the figure represents the collection time for RNA sequencing.

To obtain clues to the identity of the genes involved in *N*^*α*^-acetyl*-α-*lysine accumulation and a global view of heat-induced transcriptional changes, we performed whole-RNA sequencing of *S. halodurans* H3B36 collected from two temperature conditions, 30 °C (reference) and 42 °C (for 6 h to induce heat stress). As shown in Fig. 6, changes in the concentration of *N*^*α*^-acetyl-*α*-lysine at 42 °C versus 30 °C became apparent after 6 h. Therefore, this sampling time point for RNA sequencing was the appropriate time to study *N*^*α*^-acetyl-*α*-lysine-related genes. For each of the two treatments, three independent biological replicates were examined using RNA sequencing. After pre-processing, more than 40,000,000 clean reads (Q_20_ > 98%) were obtained from each sample. At least 96% of these clean reads from all six samples were mapped to the annotated *S. halodurans* H3B36 genome (2,778,379 bp; 2,853 predicted CDSs).

To confirm the reliability of the RNA transcriptomic data, four genes were selected for quantitative real-time reverse transcriptase PCR (qRT-PCR). Among these selected genes, orf_1589 (encoding aspartokinase), orf_1053 (encoding the Trk system potassium uptake protein TrkA) and orf_2472 (encoding a branched-chain amino acid aminotransferase) were upregulated, whereas orf_2475 (encoding the sodium/glycine symporter GlyP) was downregulated under heat stress. 16S rDNA was selected as the internal control gene to normalise the relative transcript levels. The results showed that the transcription levels of the selected genes identified using RNA sequencing and qRT-PCR followed the same trend and had a high correlation coefficient (R^2^ = 0.980), which partially validated the transcriptomic data (see Supplementary Fig. S6 online).

After screening using the cut-off criteria, 260 genes (9.11% of total) and 141 (4.94% of total) genes were downregulated and upregulated, respectively, due to the 6 h heat stress treatment (Supplementary Dataset S1). Among these differentially expressed genes, 24.7% encoded proteins of unknown function or hypothetical proteins. Eighteen genes encoding transcriptional factors were altered under heat shock (11 upregulated, 7 downregulated). To evaluate functions of the differentially expressed genes, we used the GO and KEGG databases for functional classification and enrichment analysis. A Bonferroni corrected *p*-value ≤ 0.05 was used as the threshold. Approximately 62.3% and 41.4% of the genes were related to GO and KEGG pathways, respectively. Based on the GO functional classification and enrichment analysis, 61 (15.21%), 116 (28.93%) and 73 (18.2%) significantly altered genes were involved in cellular component, molecular function and biological processes, respectively. The KEGG pathway analysis primarily grouped these genes into carbon metabolism (6.23%), the biosynthesis of amino acids (8.73%) and the ribosome (5.74%). In general, the significantly differentially expressed genes that participated in adapting to heat stress spanned various metabolic processes (carbohydrates, amino acids, nucleotides, lipids and cell wall and secondary metabolites), energy production and conversion, membrane transport, stress protection, replication and repair, transcription, translation, posttranslational modification and signal transduction.

These data suggest that heat stress triggered a global transcriptional disturbance and then generated a series of actions to adapt the host to heat stress. When cells are transferred from 30 °C to 42 °C, it is assumed that *S. halodurans* H3B36 reduces consumption and growth and increases the synthesis of cytoprotectants. This strategy could be reflected in the growth curve, the accumulation of *N*^*α*^-acetyl-*α*-lysine and the RNA-sequencing experiment. The final OD_600_ decreased and the intracellular concentration of *N*^*α*^-acetyl-*α*-lysine increased during growth at 42 °C. Moreover, the growth limitation at 42 °C was also reflected in transcription levels. Many genes related to cell growth and reproduction were downregulated, such as those involved in the glycolytic pathway and TCA cycle; genes encoding aminoacyl-tRNA synthetases, the small- and large-ribosome subunits, the ATP synthase subunits, and several key genes related to fatty acid biosynthesis; and genes linked to the translation initiation factor, the translation elongation factor and cell division.

#### Transcriptional changes of putative genes related *N* ^*α*^-acetyl-*α*-lysine biosynthesis

The transcriptomic data indicated that all 8 genes in the gene cluster probable related *N*^*α*^-acetyl-*α*-lysine biosynthesis were highly upregulated after 6 h of heat stress (Fig. 5). Their transcriptional levels exhibited 3.4- to 8.2-fold increases. Based on the HPLC quantitative analysis, intracellular *N*^*α*^-acetyl-*α*-lysine began to increase after 4 h of heat treatment and showed a notable rise after 6 h (Fig. 6). The two experiments showed a correlation between the transcription pattern of the gene cluster and *N*^*α*^-acetyl-*α*-lysine accumulation levels, suggesting that the gene cluster is likely involved in *N*^*α*^-acetyl-*α*-lysine biosynthesis from aspartate through a pathway similar to that of acetyl-dependent DAP.

However, the *N*^*α*^-acetyl-*α*-lysine gene cluster lacked an aminotransferase-encoding gene. Aminotransferase is an essential enzyme regardless of which pathway is used to synthesise *N*^*α*^-acetyl-*α*-lysine from aspartate. Searching for aminotransferases in transcriptomic data found that only two related genes, orf_2472 (encoding a branched-chain amino acid aminotransferase) and orf_2725 (encoding a histidinol-phosphate aminotransferase), were significantly upregulated under heat stress. The transcription levels of orf_2472 and orf_2725 were induced by 4.26- and 2.00-fold, respectively. Orf_2752 is in a gene cluster; all 9 genes in this cluster participate in histidine biosynthesis, and their transcription levels are increased by approximately 2-fold under stress (see Supplementary Fig. S7 online). This finding indicates the orf_2752 is more likely to be involved in histidine biosynthesis. Based on its annotation, orf_2472 participates in the metabolism of leucine, isoleucine and valine. Whereas the transcription level of other genes in the biosynthesis and degradation of leucine, isoleucine and valine were all downregulated under heat stress (see Supplementary Fig. S8 online), the opposing regulation of orf_2475 indicates that it may be an aminotransferase involved in *N*^*α*^-acetyl-*α*-lysine synthesis.

The ^13^C-labelled experiments suggest that *S. halodurans* H3B36 can convert lysine to *N*^*α*^-acetyl-*α*-lysine directly. The acetyltransferase may be the key enzyme involved in this process. Searching for acetyltransferases in the transcriptomic data, we found that only orf_1585, which encodes a 2,3,4,5-tetrahydropyridine-2,6-dicarboxylate N-acetyltransferase, was upregulated. There were three hypotheses for this result: First, the enzyme that is encoded by orf_1585 is a bifunctional enzyme that can catalyse 2,3,4,5-tetrahydrodipicolinate to N-acetyl-L-2-amino-6-oxopimelate and lysine to *N*^*α*^-acetyl-*α*-lysine. Second, the acetyltransferase involved in lysine to *N*^*α*^-acetyl-*α*-lysine conversion has a higher enzyme activity at 42 °C, and its transcription level does not significantly change. Third, the reaction from lysine to *N*^*α*^-acetyl-*α*-lysine may be a growth phase-dependent or stress-dependent chemical acetylation (nonenzymatic). Recently, many works suggest that nonenzymatic lysine acetylation on protein mediated by the reactive, high-energy metabolite acetyl-phosphate or by acetyl-CoA^44^.

From a transcriptomic perspective, *S. halodurans* H3B36 may not be the only species in the *Staphylococcaceae* to recruit lysine derivatives for cytoprotectants when facing extreme environments. In *Staphylococcus aureus*, the transcription level of five genes predicted to participate in catalysing aspartate to N-acetyl-L-2-amino-6-oxopimelate were induced significantly under NaCl stress^45^. These five genes were in the same operon, and their transcriptomic levels increased from 3.0- to 7.1-fold when *S. aureus* was grown in 2 M NaCl compared with growth in the absence of this stress. *N*^*α*^-acetyl-*α*-lysine or other lysine derivatives may accumulate in *Staphylococcus aureus* and support survival in various niches and in preserved foods, a supposition that requires further exploration.

#### Transcriptional changes of putative genes related *N* ^*α*^-acetyl-*α*-lysine transportation

**Figure 7:**
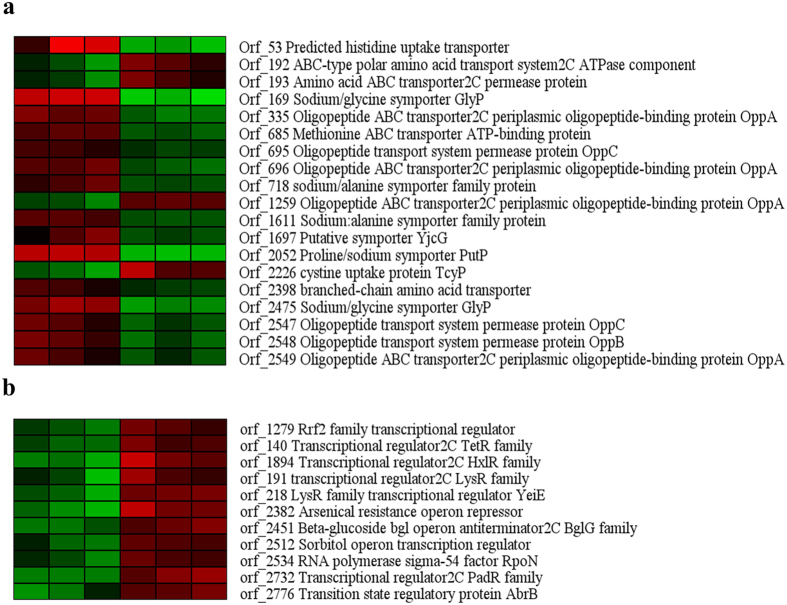
Differential transcription of genes. (**a**) genes involved in transprotation of amino acid; (**b**) genes involved in regulation. The expression profiles are presented based on log_2_FPKM. Red, upregulation. Green, downregulation. 30-1, 30-2 and 30-3 represent three duplicates originating from the 30 °C cultures; 42-1, 42-2 and 42-3 represent three duplicates originating from the 42 °C cultures.

RNA sequencing analysis showed that many genes related to amino acid transportation responded to heat stress, yet only the transcription level of orf_192, orf_193, orf_1259, and orf_2226 were upregulated (Fig. 7a). The transcription level of orf_192 and orf_193, which encode ABC-type polar amino acid transport system2C ATPase component and amino acid ABC transporter 2C permease, were upregulated 2.2-fold and 2.0-fold in the presence of heat stress, respectively. Orf_192 and orf_193 were located in an operon, which consisted of three genes. The rest of gene of this operon was orf_191, which encodes as transcriptional regulator LysR family. The transcription level of orf_191 was upregulated 2.4-fold in the presence of heat stress. The nucleotide binding domains (orf_192) and the transmembrane domains (orf_193) formed a common basic architecture of the ABC transporter. In general, the ABC transport systems of amino acids also have at least one substrate binding protein^46^,^47^, while no gene for a solute binding protein was identified in the vicinity of orf_192 and orf_193.The substrate-binding domain was not too found in the amino sequence of the enzymes encoded by orf_192 and orf_193. The transcription level of orf_1259, which encodes oligopeptide ABC transporter 2C periplasmic oligopeptide-binding protein, was upregulated 2.3-fold in the presence of heat stress. The gene of orf_1259 was an “orphan”, which no other components of the ABC transport system were located in the vicinity of orf_193. Thus, the entire operon (orf_191, orf_192, and orf_193) and gene of orf_1259 were probably involved in the heat-protection of *S. halodurans* H3B36 through providing the precursor of *N*^*α*^-acetyl*-α-*lysine.

#### Transcriptional changes of putative genes related *N* ^*α*^-acetyl-*α*-lysine regulation

The transcriptomic data also offered clues for studying the regulation of *N*^*α*^-acetyl-*α*-lysine accumulation (Fig. 7b). Several regulators belonged to the families of one-component signal transduction systems, such as the TetR family of regulators (orf_140) and the LysR family of regulators (orf_191 and orf_218), were significantly upregulated under heat stress. These genes provide good starting points for future studies on the regulation of *N*^*α*^-acetyl-*α*-lysine accumulation and biosynthesis. Sigma factors are a common regulatory mechanism. Based on its genome annotation, *S. halodurans* H3B36 possesses only three putative sigma factors: RpoD (orf_1417), SigB (orf_2321) and RpoN (orf_2534). In our study, only orf_2534 was significantly altered after exposure to 42 °C for 6 h. The transcriptomic level of this gene increased 2-fold under heat stress. RpoN was first reported to regulate the transcription of genes related to nitrogen assimilation in *Escherichia coli*^48^. Subsequent reports in other organisms have indicated that RpoN is involved in the functional regulation of diverse processes, including carbon metabolism, flagella biosynthesis, arsenite oxidation, and even resistance to stresses, such as high osmotic pressure, starvation and acid^49^,^50^,^51^,^52^,^53^,^54^. In *Geobacter sulfurreducens*, the overexpression of RpoN can inhibit growth and induce the upregulation of genes involved in stress responses^54^. This report corresponds well with our results. In our study, the growth rate and final density of *S. halodurans* H3B36 were both decreased in the heat treatment condition. When we collected cells after 6 h, the OD_600_ under the optimal temperature and heat treatment conditions were 2.35 and 1.61, respectively. Additionally, the concentration of the putative cytoprotectant *N*^*α*^-acetyl-*α*-lysine was increased. The transcriptomic data also supported the same conclusion. Heat stress caused many genes related to cell reproduction and division to be downregulated and several heat-protection genes to be upregulated. Our study suggests that the RpoN regulon may be involved in the regulation of accumulation of *N*^*α*^-acetyl*-α-*lysine, and critical to conferring heat stress tolerance and survival to *S. halodurans*.

## Conclusion

In conclusion, our work showed that *N*^*α*^-acetyl-*α*-lysine accumulates and probably acts as a thermolyte in *S. halodurans* H3B36. The concentration of this probable thermolyte increased by approximately 3-fold under heat stress. We also confirmed that *N*^*α*^-acetyl-*α*-lysine synthesis is induced by heat stress. This strain possesses a *de novo* synthesis pathway for *N*^*α*^-acetyl-*α*-lysine that starts with aspartate and continues through lysine. Genes that may be involved in the accumulation and biosynthesis of *N*^*α*^-acetyl-*α*-lysine were also identified through RNA sequencing. Furthermore, the transcriptome reported here also contributes to understanding the regulatory mechanisms of *S. halodurans* H3B36. The RpoN regulon may be critical to conferring heat stress tolerance and survival to *S. halodurans*. However, our jobs only scratched the surface on the role, genes involved in accumulation and biosynthesis, and regulation of *N*^*α*^-acetyl-*α*-lysine in *S. halodurans* H3B36, and more hard proofs need to be presented in the future, like construction of mutants lacking the biosynthetic genes. Unfortunately, experiment of gene knockout still could not be achieved in the *S. halodurans* H3B36 right now. Overall, this work presented prelimimary studys on *N*^*α*^-acetyl-*α*-lysine in *S. halodurans* H3B36, and subsequent research may lead to provide a further evidence for the hypotheses of *N*^*α*^-acetyl-*α*-lysine in *S. halodurans* H3B36 and the discovery of new enzymes and enable the biotechnological production of *N*^*α*^-acetyl-*α*-lysine.

## Materials and Methods

### Strains and growth conditions

*S. halodurans* H3B36 was grown in GMH medium supplemented with different concentrations of NaCl or in MM medium, as noted. Medium pH was adjusted with HCl or NaOH. GMH medium contained 5 g/L casamino acids, 5 g/L yeast extract, 4 g/L MgSO_4_·7H_2_O, 2 g/L KCl, 0.036 g/L FeSO_4_·7H_2_O, 0.36 mg/L MnCl_2_·7H_2_O, and 60 g/L NaCl at pH 7.0 (adjusted with 1 M NaOH). MM medium contained 100 mmol/L KH_2_PO_4_, 75 mmol/L KOH, 15 mmol/L (NH_4_)_2_SO_4_, 1 mmol/L MgSO_4_, 0.0039 mmol/L FeSO_4_, 22 mmol/L D-glucose, and 40 g/L NaCl at pH 7.0 (adjusted with 1 M HCl). Except as noted, all experiments were performed in conical flasks shaken at 210 rpm and at 30 °C. Growth was monitored by measuring turbidity at 600 nm. The effects of salt stress on the accumulation of *N*^*α*^-acetyl-*α*-lysine were examined in GMH medium with 2% NaCl or 14% NaCl. The effects of heat on the accumulation of *N*^*α*^-acetyl-*α*-lysine were examined in GMH medium at different cultivation temperatures. Growth and *N*^*α*^-acetyl-*α*-lysine accumulation were monitored during the experiments.

### Extraction of compatible solutes

Cells were harvested by centrifugation at 8,000 × *g* for 10 min. The cell pellet was washed twice with an isotonic solution containing the appropriate NaCl concentration and was subsequently centrifuged at 8,000 × *g* for 10 min. Then, the supernatant was removed, and the pellet was freeze-dried, weighed and extracted using a modified Bligh and Dyer technique^55^. The extract was processed overnight by vigorously stirring the material with four volumes of methanol:chloroform:water (10:5:3.4 by vol). Then, 1.3 volumes each of chloroform and water were added to the mixture. After vigorous shaking (1 h), centrifugation (5,000 × *g*, 30 min) was necessary to promote phase separation. The aqueous top layer, containing the compatible solutes, was recovered and prepared for further experiments.

### HPLC analysis

An appropriate amount of acetonitrile was added to each sample of the cell extract to obtain the same ratio of acetonitrile as the mobile phase for HPLC analyses. Then, the mixture was filtered through a 0.22 μm membrane and analysed on the Agilent 1200 HPLC system. Ten microlitres of each sample were analysed using a Nucleosil 100-5 NH_2_, 250 × 4 mm column (with a 3 μm pore size) (MACHEREY-NAGEL, Germany) at 30 °C with an acetonitrile/water (70:30, v/v) solution as the mobile phase at a flow rate of 1.0 mL/min. Elution was monitored using a DAD at 210 nm and RID. The retention times and concentrations of compatible solutes were determined using commercially available standards.

### Preparation of the unknown compatible solute

The unknown compatible solute reflected in the HPLC and NMR spectra was isolated and purified using a semi-preparative chromatography column (Nucleosil 100-5 NH_2_, 250 × 10 mm column with a 5 μm pore size) with an acetonitrile/water (70:30, v/v) solution as the mobile phase at a flow rate of 5.0 mL/min. The separated compound was concentrated and re-purified with the column to improve purity. Fractions containing the compound were pooled and freeze-dried.

### LC-MS and elemental analyses

An Agilent 1200 HPLC/6520 Q-TOF MS was used to determine the molecular mass and formula of the unknown compatible solute in the cell extract. The sample preparation procedure and HPLC conditions used were the same as described above. The effluent was introduced into the mass spectrometer directly and analysed in electrospray ionisation mode (ES+) after the liquid chromatography column (Nucleosil 100-5 NH_2_, MACHEREY-NAGEL). The elemental analysis, which was used to determine the C:H:N ratio, was performed in a Flash EA 1112 (Thermo, US) using the purified target compound.

### NMR analysis

The purified compound from the cell extract was dissolved in 0.5 mL of D_2_O and placed in 10-mm-diameter NMR tubes for the NMR analysis. The ^1^H-NMR, ^13^C-NMR and 2D-NMR (heteronuclear multiple bond coherence, HMBC) spectra were measured on a Bruker AVANCE III 500 spectrometer with a 5 mm CPPBBO probe head to determine the preliminary structure and confirm the identity of the unknown compatible solute. 3-(trimethylsilyl)-2,2,3,3-d_4_ propionic acid sodium salt (abbreviated as TMSP) served as the internal reference.

### Heat shock experiment and total RNA extraction

*S. halodurans* H3B36 was cultured in GMH medium containing 6% (w/v) NaCl at 30 °C to mid-exponential phase (an OD_600_ range of 0.5 to 0.6). Then, the culture was split into six 250 mL conical flasks, three were continuously cultured at 30 °C and three were cultured at 42 °C, all shaken at 210 rpm. The intracellular *N*^*α*^-acetyl-*α*-lysine concentrations of the six cultures were measured using HPLC after heat shocking for 2, 4, 6, 8, or 20 h. The total RNA collected at 6 h in all 6 cultures was used for RNA sequencing. The pellets were rapidly harvested by centrifugation (10,000 × *g* for 1 min) and frozen in liquid nitrogen. Total RNA was isolated using grinding in liquid nitrogen combined with TRIzol (Invitrogen, USA) extraction according to the manufacturer’s instructions. The quantity and quality of the total RNA was assessed with a NanoDrop (Thermo Fisher Scientific, USA), a Bioanalyser 2100 (Agilent, USA) and agarose gel electrophoresis.

### Paired-end index library preparation and sequencing

From each of the six samples, 1 μg of RNA was used for library preparation. Paired-end index libraries were constructed using the manufacturer’s protocol (NEBNext® Ultra™ RNA Library Prep Kit for Illumina®). The strands of cDNA were synthesised using ProtoScript II Reverse Transcriptase and the Second Strand Synthesis Enzyme Mix and then purified using the AxyPrep PCR Clean-up kit (Axygen, USA). End repair, 5′phosphorylation and dA-tailing were completed in one reaction with the End Prep Enzyme Mix. After ligation to adaptors with a “T” base overhang, the adaptor-ligated DNA was selected to recover approximately 400 bp fragments using the AxyPrep Mag PCR Clean-up kit (Axygen, USA). Next, each sample was bound to the surface of the flow cell to enable bridge PCR amplification for 11 cycles using P5 and P7 primers, followed by multiplexing. The PCR amplicons were then purified using the AxyPrep Mag PCR Clean-up kit (Axygen, USA), validated on an Agilent 2100 Bioanalyser, and quantified by real-time PCR (Applied Biosystems, USA) and Qubit. The obtained libraries were sequenced with a HiSeq 2500 machine (Illumina) using a 2 × 125 paired-end (PE) configuration; image analysis and base calling were conducted by the HiSeq Control Software (HCS) + OLB + GA Pipeline 1.6 (Illumina) on the HiSeq instrument.

### Transcriptomic data analysis

The sequences were analysed using the NGS QC Toolkit (v2.3). Raw reads were pre-processed to remove adaptors, low-quality bases and trimmed-reads shorter than 75 bp. Clean reads were separately mapped to the genomic sequence and reference genes of strain H3B36 using Bowtie 2 (v2.1.0) with the default parameters. FPKM (fragments per kilo bases per million reads) normalisation was used to compute the gene-expression values. The *P-value* was adjusted using the Benjamini false discovery rate (FDR). Genes with fold changes >2 and adjusted *P-*values ≤ 0.05 in FPKM between the two conditions were defined as differentially expressed. The Multiexperiment Viewer (MeV) in the TM4 microarray software suite was used for cluster analysis and heat maps.

### qRT-PCR

The RNA samples were treated with RQ1 RNase-Free DNase (Promega, USA) to eliminate residual genomic DNA and reverse transcribed using the PrimeScript® RT Reagent Kit (TaKaRa, Japan), and the qRT-PCR reactions were performed on a Bio-Rad CFX96 Real-Time PCR System using the SYBR Green EX Taq mix (TaKaRa, Japan) with the kit-recommended two-step standard cycling method. Every qRT-PCR was performed in triplicate with a volume of 25 μL and repeated more than three times under the same conditions in a separate experiment. For the relative quantification of specific genes from different transcripts, the cycle threshold (*C*_*T*_) method was used to calculate fold changes in expression. The 16S rRNA gene was chosen as the reference gene to normalise the variability in expression levels. The orf_1053, orf_1589, orf_2472 and orf_2475 genes were selected to verify the RNA sequencing results. Primer Premier 5 was used to design the forward and reverse primers for each selected gene. The efficiency of each primer pair was calculated using the standard curves of 10-fold serial dilutions of genomic DNA by the Bio-Rad CFX Manager.

### ^13^C-labelling experiment

Cell cultures of *S. halodurans* H3B36 were grown at 30 °C in GMH medium, GMH medium plus 5 mmol [1-^13^C] lysine (Cambridge Isotope Laboratories, USA), or GMH medium plus 5 mmol [1,4-^13^C] aspartate (Cambridge Isotope Laboratories, USA). Once the end of the exponential growth phase was reached, the cells were harvested. Then, the compatible solutes were extracted, and *N*^*α*^-acetyl-*α*-lysine was isolated. ^13^C NMR was used to identify *N*^*α*^-acetyl-*α*-lysine.

## Additional Information

**How to cite this article**: Jiang, K. *et al.* Identification of *N*α**-acetyl-*α*-lysine as a probable thermolyte and its accumulation mechanism in *Salinicoccus halodurans* H3B36. *Sci. Rep.* **5**, 18518; doi: 10.1038/srep18518 (2015).

## Supplementary Material

Supplementary Figures

Supplementary Dataset S1
