# Supplementary material for: Identification of Nα-acetyl-α-lysine as a probable thermolyte and its accumulation mechanism in Salinicoccus halodurans H3B36
Source: Sci Rep. 2015 Dec 21;5:18518. doi: 10.1038/srep18518 (PMC4685198; doi:10.1038/srep18518)
Supplement: Supplementary Figures [file srep18518-s1.pdf]

Identification of N^{α}-acetyl-α-lysine as a probable thermolyte and its accumulation mechanism in *Salinicoccus halodurans* H3B36

Kai Jiang, Yanfen Xue, Yanhe Ma

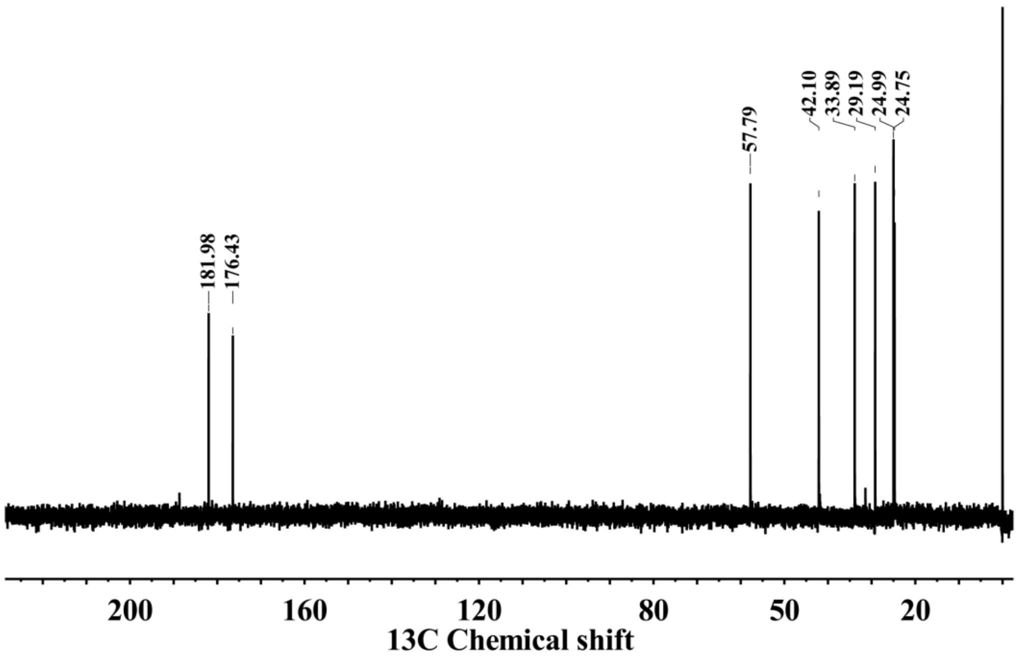

Figure S1. Natural abundance ^{13}C spectrum (recorded in D2O as a solvent) of the purified unknown compound. TMSP was used as the internal reference.

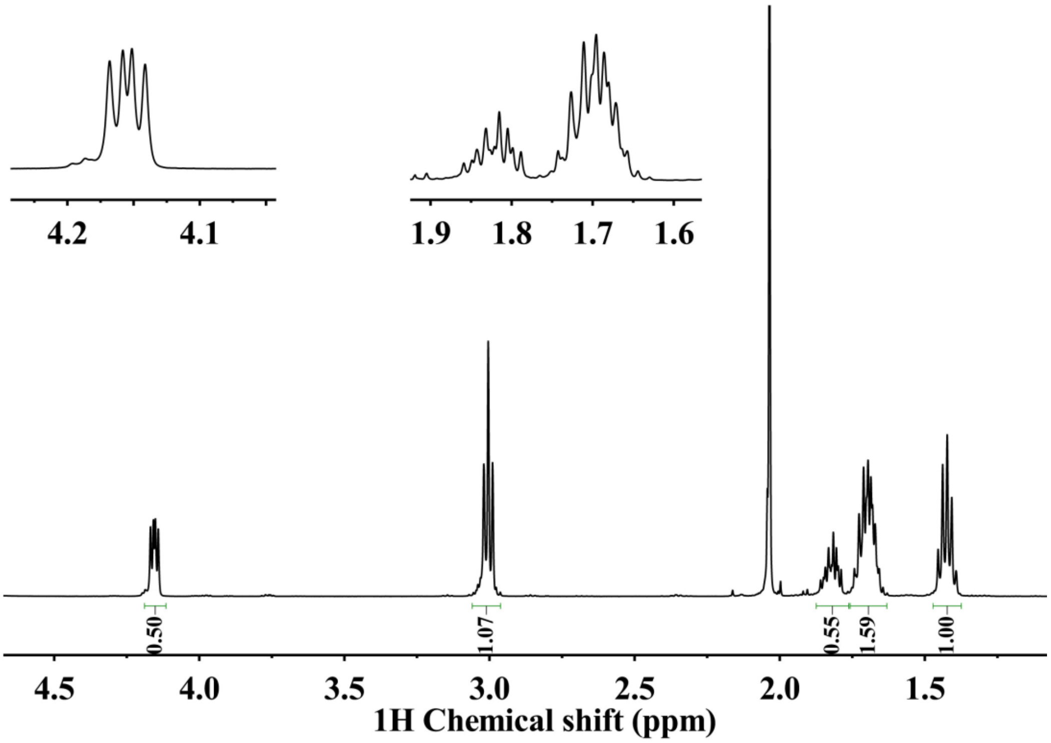

Figure S2. 1D NMR-^{1}H spectrum (recorded in D2O as a solvent) of the purified unknown compound. TMSP was used as the internal reference.

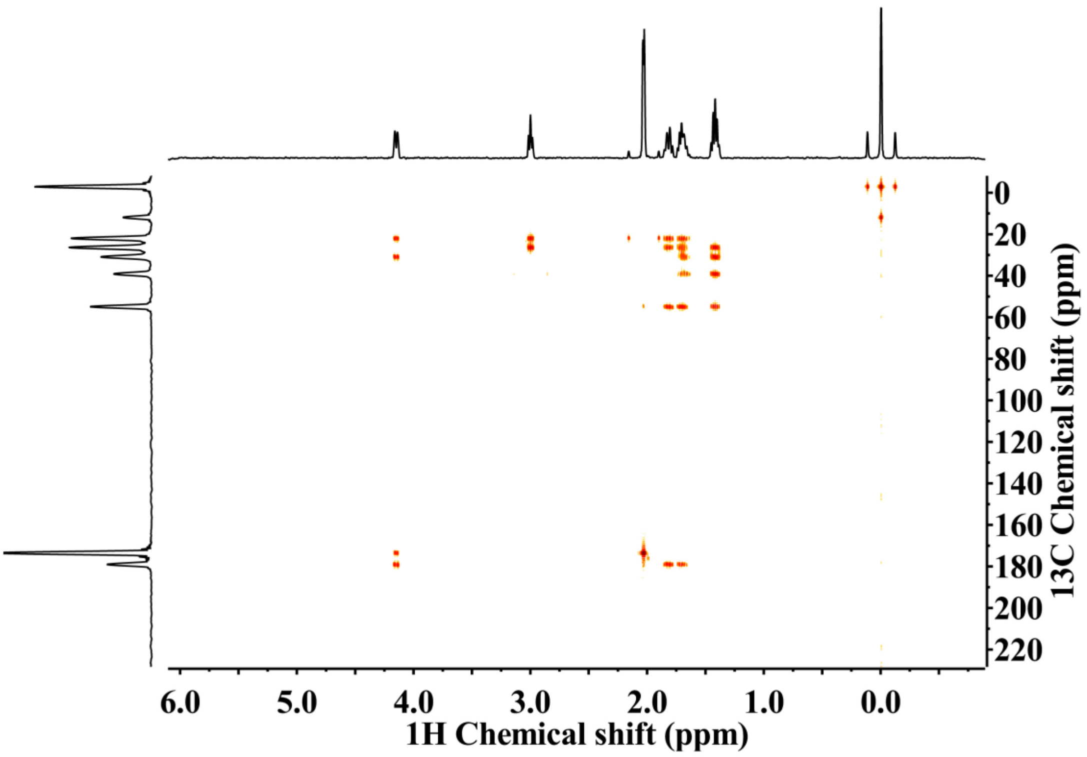

Figure S3. 2D NMR-HMBC spectrum (recorded in D2O as a solvent) of the purified unknown compound. Cross spots in the spectrogram represent correlation between carbon atoms and protons separated by two or three bonds. TMSP was used as the internal reference.

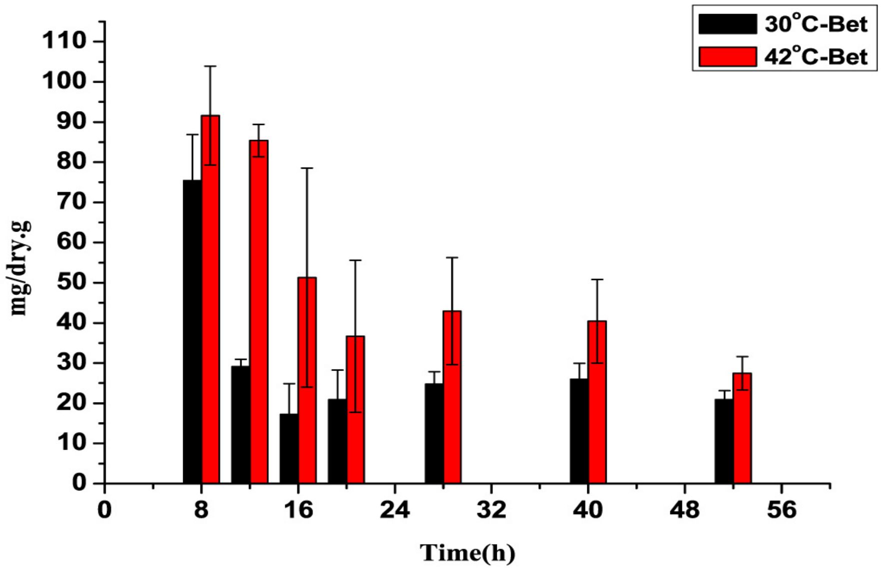

Figure S4. Intracellular amounts of Glycine betaine in *S. halodurans* H3B36 under heat stress. Intracellular amounts of glycine betaine were calculated based on cellular dry weight. 30℃-Bet and 42℃-Bet represent the amount of Glycine betaine of cells cultured at 30℃ and 42℃, respectively.

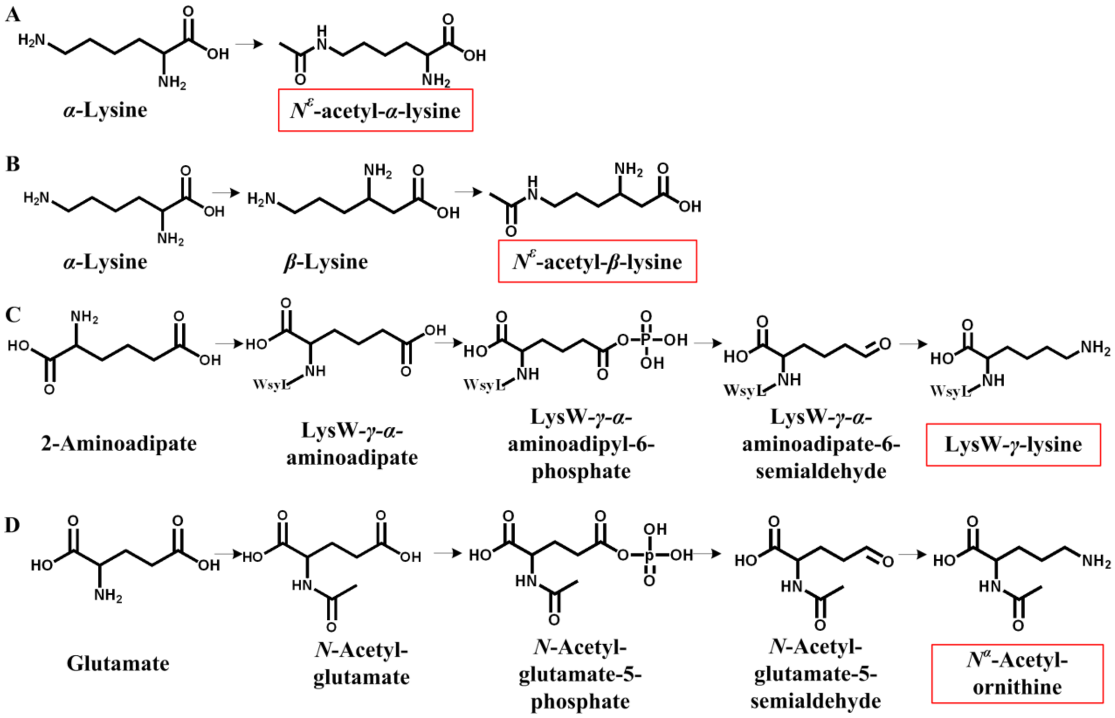

Figure S5. The pathway of N^{ε}-acetyl-*α*-lysine, N^{ε}-acetyl-*β*-lysine, LysW-γ-lysine, and N^{α}-acetyl-ornithine.

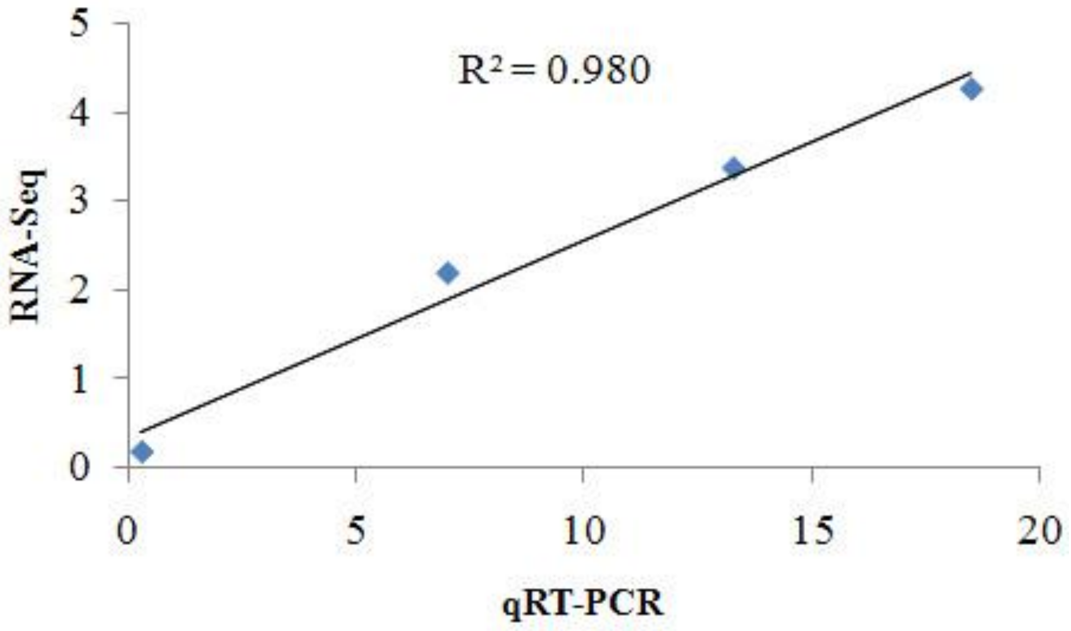

Figure S6. Validation of the RNA sequencing results by qRT-PCR.

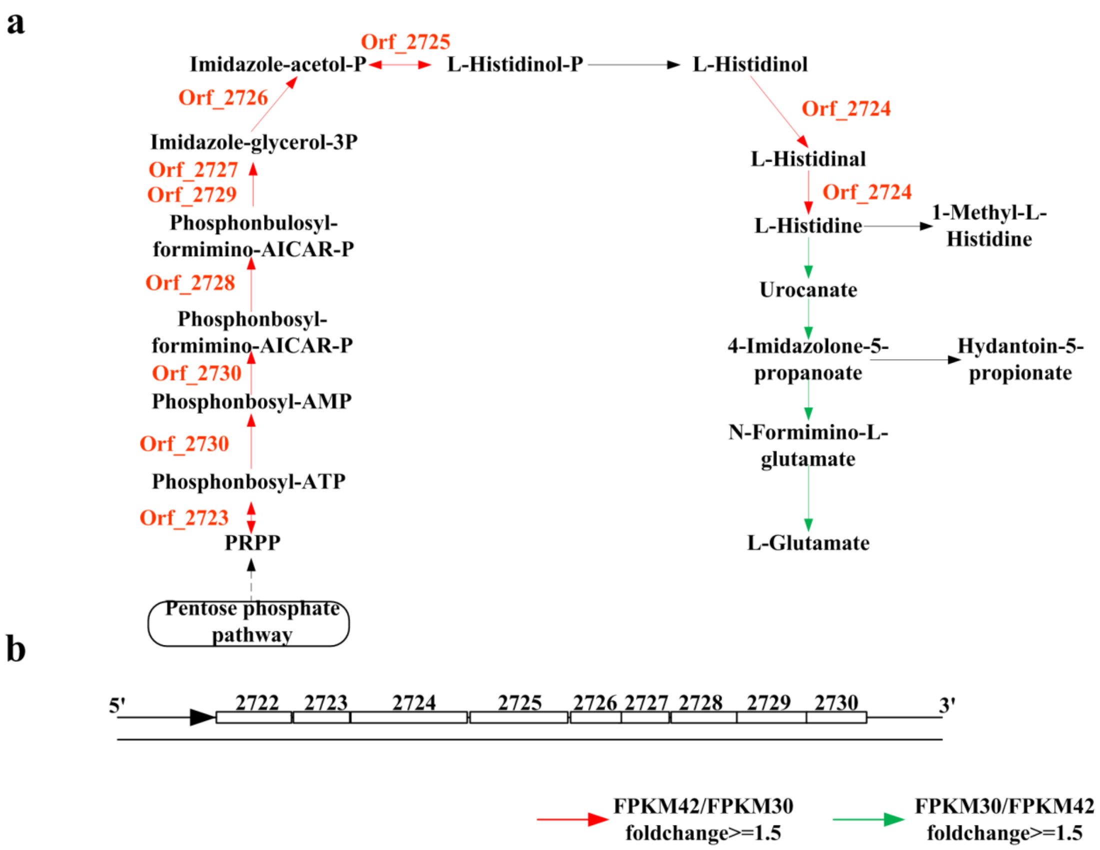

Figure S7. Differential transcription of genes related to orf_2725 in *S. halodurans* H3B36. Red, up-regulation. Green, down-regulation.

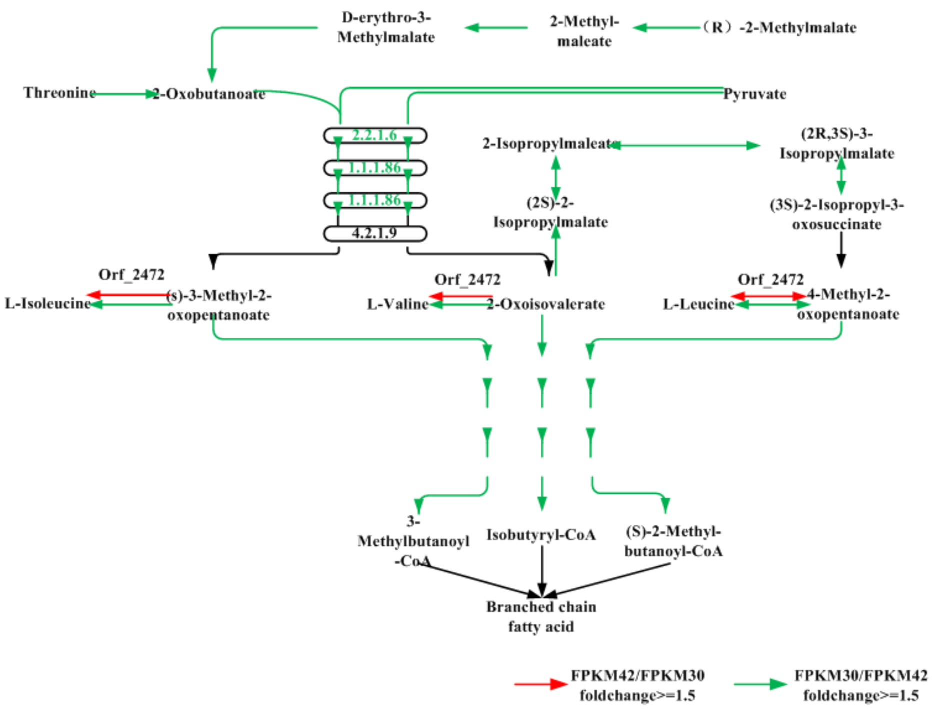

Figure S8. Differential transcription of genes related to orf_2472 in *S. halodurans* H3B36. Red, up-regulation. Green, down-regulation.
